# Converting a Cough Counter into a Cough Monitor: A Way Forward?

**DOI:** 10.3390/medsci14020327

**Published:** 2026-06-17

**Authors:** Albertus C. den Brinker, Michael G. Crooks, Alyn H. Morice

**Affiliations:** 1 Independent Researcher, NL-5708 DJ Helmond, The Netherlands; 2 Centre for Clinical Science, Hull York Medical School, University of Hull, Cottingham HU16 5JQ, UK; michael.crooks@nhs.net (M.G.C.); a.h.morice@hull.ac.uk (A.H.M.)

**Keywords:** COPD, exacerbation, alert, cough, monitoring, reliability metric, eHealth

## Abstract

**Background/Objective**: To identify respiratory pathology, automated cough counting is frequently proposed. A trial validating an early warning system for exacerbations in chronic obstructive pulmonary disease (COPD) patients was recently concluded successfully. This paper aims to review the critical design choices for converting a cough counter into a patient-friendly continual cough monitor. Furthermore, it provides a basis for a practical reliability metric for continual cough monitoring. **Methods**: Design choices made in the development of a cough-based alert mechanism called XACT are discussed. A practical approach for reliability assessment is outlined based on cough counts, day-to-day variation and specificity data. **Results**: In post hoc analysis, it is shown that the described approach enables differentiation between high-quality cough estimates and less reliable data. The approach is used to underpin an earlier cohort subdivision into patients with and without increased cough during exacerbation. **Conclusions**: The validated alert mechanism has various patient-oriented design choices (unobtrusiveness, privacy-preserving). The examples illustrate how to screen for potential issues in automated cough count data without resorting to laborious annotation. It creates a practical basis for confidence metrics of medical inferences made from cough data, e.g., exacerbation forecasts. The proposed concepts need further validation.

## 1. Introduction

With the rise of artificial intelligence (AI), automated cough identification has received significant attention [1,2,3,4,5,6,7]. Cough identification has been proposed for various respiratory diseases including chronic obstructive pulmonary disease (COPD), asthma, bronchiectasis, cystic fibrosis, and, of course, chronic cough, with applications comprising (differential) diagnosis, intervention testing, continual health status inference and patient education.

When cough identification is applied to diagnostics, one or more patient coughs are collected, and an automated system has been claimed to classify the underlying lung disease (normal, COPD, asthma, etc). This line of research has a long history yet lacks compelling evidence [4] and has not resulted in any U.S. Food and Drug Administration (FDA) approved system. In this paper, we only consider cough counting systems.

In intervention testing, the hypothesis is that an intervention (e.g., a drug) will have a significant effect on cough behaviour. The outcome is cohort-oriented: in the mean, a change needs to be shown. Adherence of each and every participant to the monitoring protocol is not a prime issue: non-adhering patients are often excluded from the analysis of the data. With less patients, the expected average difference remains the same, only the reliability of the observed change decreases. Patients need not to be monitored continuously; it may suffice to show that the intervention had positive effects after some predefined time. This means that the counting is expected to be short in duration. The cohort may be subdivided in the analysis to show effects within one or more subgroups.

The scenarios of continual health status inference and patient education have much in common and are the topic of this paper. In the first case, the target is to infer a change in the patient’s health status (or its forecast), while in the second one it is insights into respiratory health (from the cough biomarker) in relation to environmental factors or behaviour. These objectives are more demanding than that of intervention testing. Each patient provided with a monitor is expecting effect from the monitoring. The monitoring is oriented on patients with frequent cough and is long-term (years) except for the case of verifying convalescence/rehabilitation. For a generic cough classifier, this means that the average number of false positives (FPs) must be substantially below that of true positives (TPs) within any given acoustic environment. The worst-case scenario is a low number of coughs within a highly adverse acoustic environment, raising the likelihood that FPs will outnumber TPs. Monitoring over a prolonged period of time requires patient adherence and therefore device unobtrusiveness, and low additional burden (preferably none) is critical. With this in mind, while a portable or wearable device may be preferable for interventional tests, they are less appropriate for long-term patient monitoring.

People with frequent cough have highly variable day-to-day cough counts, with a coefficient of variation (CV) of 0.3–0.4 [8,9]. In line with the fact that daytime activities and environments fluctuate much more than night-time behaviour and the bedroom environment, the CV for 24 h cough counts is higher than that for night-time cough counts. A lower CV implies clearer trends (less noise) and simpler inference rules from trending data. The caveat is that the number of night-time coughs is relatively low.

Cough counts have been proposed as a means to improve the understanding of patient-specific relations between respiratory health status and activities or environmental factors. In such an educational proposition but also for adoption by clinicians, interpretability of the data is a key factor (i.e., detection and depiction of cough trends). Therefore, if AI is used to infer health status from cough counts, it has to be explainable (XAI [10]), e.g., translatable in something akin to simple rules. The use of AI approaches to predict exacerbations from cough trends requires many instances of exacerbations across a large population, making it problematic to design and parameterise at least in a development phase. Both development and deployment benefit from simplicity.

Various modalities have been proposed to capture cough. Microphones are the most common sensors used but accelerometers [11,12,13], and combinations of modalities [14,15] have also been considered. The desire for an unobtrusiveness system favours acoustic monitoring over a body-worn accelerometer. Combined bedtime monitoring using sound and accelerometer signals from the bed has also been proposed [16]. Such a system would likely be highly dependent on sensor position and bed-type, potentially limiting its application. In this paper, only the sound modality is considered.

Two examples where cough monitoring would be appropriate are pediatrics and COPD. In this paper, we concentrate on COPD. COPD exacerbations are associated with morbidity and mortality in COPD [17,18] and frequently lead to unscheduled healthcare resource utilisation, contributing to pressure on healthcare systems. Cough and sputum production are reported by 60–80% of COPD patients [19,20] and are increased at the time of exacerbation [21,22,23,24]. It has been suggested that early interventions in COPD are effective [25]. This makes cough monitoring of COPD patients an interesting option as an aid in early detection and to facilitate early treatment.

Much research has been dedicated to automated cough classifiers. Unfortunately, most studies have the cough detection performance in traditional classification terms as prime objective instead of a clinically meaningful, patient-centred endpoint. Improved cough detection is an asset but, given an application, a secondary goal only. Research investigating the impact of clinical applications of automated cough counting (e.g., for early detection of acute exacerbation of COPD (AECOPD)) on medical benefits, such as quality of life, healthcare resource use, and cost are needed [5,6].

In a series of papers, it has been shown that the requirements for long-term continual monitoring can be met for AECOPD prediction. Night-time monitoring and a personalised cough classifier were proposed to deal with the large differences in cough (prevalence and cough character) and acoustic environments among patients [26]. A rule-based alert system was designed to support clinical adoption and educational purposes [8] and an off-body small device on the bedside created a burden-free system [27]. A stratification method was developed to identify the COPD cohort that can be effectively served [28]. An extended rule set for improved performance was proposed and tested in a post hoc analysis. The result [29] indicated sensitivity of 86% and positive predicted value in the range 65–78% for detecting an impending exacerbation. The system is called XACT as an acronym for Explainable Alert from Cough Trends.

Due to its design choices, the cough monitor was perceived as burden-free and raised no concerns with participants [27]. In [29], it was argued that the cough trends and rule-based alerts are interpretable by patients and caregivers, and therefore could become an element within an educational platform. To strengthen such an approach, a reliability metric quantifying the trustworthiness of the cough data would be helpful. Therefore, we present a practical approach to a reliability metric based on data collected in the trial. The developed reliability metric is therefore an empirical rule. The approach uses knowledge of the (current) number of coughs, the generic knowledge of day-to-day variation of night-time cough counts for stable COPD patients, and the specificity of the personalised cough classifier. By various examples, it is demonstrated how cough count trends and (coarse) false positives estimates indicate potential issues in the collected data. One of the examples can be seen as an addendum to [28]. Patients were subdivided into those having increased cough during exacerbation and those without. This subdivision was created based on correct exacerbation identifications of the tested alert system and visual inspection of the cough count trends graphs. This subdivision led to a system for patient stratification preventing monitoring of patient where cough is not a relevant biomarker. In principle, however, the cause of absence of elevated cough count might be twofold: either the patient does not have an elevated cough (i.e., cough counts stay within the normal day-to-day variation) or the increase in the coughs is masked by a large amount of false cough detections. We rechecked the data for indications of this latter unintended situation. This case highlights the use of the knowledge of cough counts, day-to-day variability, and effects due to limited specificity. Due to the privacy-preserving set-up of the trial, possibilities for full statistical analysis are limited; the present study focuses on concepts and demonstration of feasibility.

## 2. Materials and Methods

### 2.1. Data Collection

The analysed data stem from a prospective longitudinal study of continual cough monitoring in COPD patients. The study participants were monitored for 12 weeks and asked to continue for a further 12 weeks if no exacerbation had occurred during the first observation period. A research nurse visited patient’s home each month for device inspection, collection of questionnaire data, and creation of incident report forms. Inclusion criteria included a clinical diagnosis of COPD according to the NICE guidance [30], two or more moderate and/or severe exacerbations of COPD in the previous year, and a smoking history of 10 packyears or more. Patients were excluded in case of significant comorbid medical or psychological conditions affecting the cough frequency. The principal investigator also checked participants’ psychological conditions to ensure ability to comply with trial procedures. To mitigate investigator bias, the trial was executed in a double-blind fashion meaning that exacerbations were identified without knowledge of the objective cough counts and objective cough processing and alert generation were done without knowledge of medical data from the patient. The cohort was set at 40 with baseline demographics presented in [27] and data from 32 participants were suitable for cough count analysis. The study was reviewed and approved by the Internal Committee Biomedical Experiments of Philips Research and the North East-York Research Ethics Committee, United Kingdom Health Research Authority (REC Ref.: 21/YH/0203), with informed consent obtained from all participants involved in the study.

### 2.2. Data Processing

**Figure 1:**
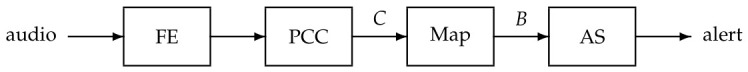
Cough-based acute exacerbation of COPD (AECOPD) alert system XACT consisting of a feature extractor (FE), a personalised cough classifier (PCC), a mapping of the cough count to the B-scale (Map) and a rule-based alert system (AS). The output of the classifier is the cough count indicated as *C* and and the mapped cough count is denoted as *B*.

The cough monitor was a stationary system placed in the bedroom of the patients and operated between 9 p.m. and 9 a.m. The processing of the alert system is shown in Figure 1 and consists of 4 subprocesses. In the first process, audio features were extracted. The use of a feature-based approach in the home of the patient with only features or derived data being transmitted ensures the privacy of the patient. In our trial set-up, a limited number of features were accompanied with a 1 s audio snippet. This provided means for checking the acoustic environment and to acquire data to train the classifier while protecting participants’ privacy.

The feature extraction receives an 8 kHz sampled audio signal from a measurement microphone (Dayton IMM6, Dayton Audio, Sprongboro, Ohio) and detects an audio transition with a method based on linear prediction analysis. When a transition is detected, a fine-grain transition position search is executed and spectral parameters (MFFC-like) around the transition are calculated as well as several time-domain ones (energy before and after transition and density of acoustic events). A variant of Mel frequency cepstral coefficients (MFCCs) was used with band filters having equidistant spacing on an equivalent rectangular bandwidth (ERB) scale [31]. In total, it means that a time stamp is generated and a very limited number of features is available for classification.

Sound samples of 1 s length around detected transitions are temporarily stored during intervals of 15 min. The sound snippets over this interval are a random subset with a predefined maximum number. The selection is slightly biased towards the louder signals to attain a more balanced set. The balancing and loudness bias also eases the annotation task. Choices in this data collection format were based on earlier studies.

For each patient, a classifier was trained using an extreme gradient boosted decision tree classifier (XGBoost 2.1.1 with binary:logistic classifier, python implementation 3.10) using the annotated snippets from the first days. Using around 200 coughs per patients resulted in robust classification model. More details on the training method and performance characteristics can be found in [32]. The trained personalised cough classifier was operated on all sound features.

Per acoustic event (represented by a feature set), the classifier produces a number in the range 0 to 1, usually interpreted as a ‘probability’, which is translated into the decision: ‘cough’ or ‘non-cough’ using a threshold. A threshold of T=0.9 was used uniformly over all patients. Setting a high threshold lowers sensitivity but ensures a high specificity needed in view of a generally low cough prevalence [26] and provides resilience against changes in the acoustic environment of the patient. The fact that the number of coughs may be systematically underestimated due to a low sensitivity is not prohibitive: for monitoring (trending) the changes in cough count is the key information, and not the absolute level.

The caveat would appear here that exacerbations of patients with a low cough prevalence are at higher risk of going unnoticed. However, it is questionable if patients with low cough prevalence are served by an alert system based on the cough biomarker. Analysis [28] suggested that the cough biomarker is best suited for the older and more severe COPD patients. A reason for this finding has still to be determined, but it may be due to the skewed, heavy-tailed distribution of cough counts in low cough prevalence situations. Effective means for exacerbation prediction would in that case be reserved to other biomarkers.

The night-time cough count, denoted as *C*, was represented on the B-scale introduced in [8] which is a logarithmic mapping given by

(1) $$B=\alpha \log_{10}\left\{1+\beta C\right\}$$

with constants α=3.45 and β=0.04. This mapping is based on the natural day-to-day variation of night-time cough and creates a scale where differences are interpretable independent of level. Therefore, the mapping facilitated the design of a generic alert mechanism. In fact, the time series *B* is the input to the alert mechanism and therefore the quantity of interest. In [28], it was shown that distance between the median and third quartile is approximately 0.35 B independent of person. For the higher cough counts, the distance between median and first quartile was modelled as 0.3 B (see Appendix A). These ranges are referred to as the generic quartile distances.

### 2.3. Alert Mechanism

In [8], a rule-based alert mechanism was proposed, which was later extended in [29]. The rule-based mechanism is instrumental to being able to parameterise and test the system with only a limited number of exacerbations. Furthermore, it provides the clinician, caregiver and/or patient with an intuitive and easily verifiable explanation for the presence/absence of an alert.

In short, the alert mechanism receives the mapped cough count *B* and processes this in two parallel branches. One branch checks for fast consistent increase and a steep rise over a few days. The other branch considers the last fortnight to derive a baseline and checks if the (smoothed) cough count is far above the baseline twice in the last 3 days. The temporal smoothing on the cough count in the alert mechanism is executed by a (causal) first-order recursive filter. Its recursive parameter is a compromise between amount of noise-suppression and the inevitable delay and was set to the same value as used previously: $p_{t}=0.5$ [32]. All settings in the alert mechanism are fixed and not personalised like in the cough classifier.

### 2.4. False Positives

Note that the alert system operates only on the time series *B*. It is blind to whether the data in the time series are reliable. To characterise data quality issues due to a potential excessive number of false positives, a coarse estimate of the number of false positives is created and compared with the cough count. The coarse estimate of the false positives is based on two assumptions: i. the number of coughs is much lower than the number of acoustic events that was classified within each night; and ii. the specificity number obtained in the training testing/phase of the personalised classifier is representative for its deployment phase. With these two assumptions, the number *F* of FPs in the deployment phase is approximated by

(2) $$F=\left(1-s_{p}\right)N_{nc}\approx \left(1-s_{p}\right)N_{ae}$$

where $s_{p}$ is the specificity, $N_{nc}$ the number of non-coughs and $N_{ae}$ the number of acoustic events during the monitoring interval. The coarse estimate for the false positives for each night is proportional to inputs to the classifier. It can be viewed as a reality check: is there a chance that the number of coughs indicated by the system is heavily polluted by the number false positives due to a high number of acoustic events and/or low specificity? To illustrate the added insights and potential use of this empirical approach, we present and discuss traces of cough count and expected FP.

### 2.5. Patient Stratification

**Figure 2:**
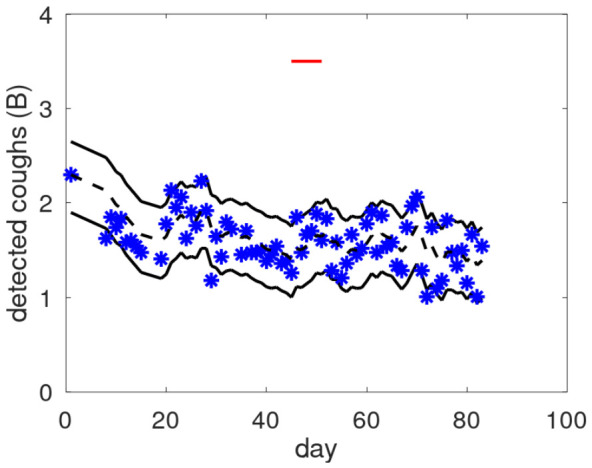
Cough counts (blue asterisk), smoothed trend (black dashed line), and quartiles of day-to-day variation [28] (black solid lines). The red horizontal line indicates the exacerbation period. The alert mechanism did not create an alert for this patient [27], nor does the data themselves reveal clear excursions outside of the normal day-to-day variation. The black solid lines are calculated by smoothing the raw data [29] and adding and subtracting to this the validated generic quartile distances [32].

In [28], two subgroups were created from the patients with an exacerbation during the monitoring period. These were patients labelled as with or without an increase in cough count during exacerbation. Seven patients were identified as having cough count graphs without increased cough counts during exacerbation. An example of such data is shown in Figure 2. The night cough counts were smoothed with the (causal) filter present in the alert mechanism (Section 2.3) and a band is created by adding the generic quartile ranges (Section 2.2).

Since raw cough counts do not consistently but only occasionally fall outside of this band, it illustrates that the cough data of this patient is not a marker for an exacerbation. The use of average cough count, knowledge of normal day-to-day variation, and the expected number of FPs allows to check this in a more numerical setting for all seven patients by comparing average, spread and an estimate of the false positives.

Details of this process are as follows. The statistics of the (night-time) cough count over the monitoring period were created and represented by the median, denoted as $C_{m}$. From the data used for training the cough classifier, the specificity was calculated. Multiplying the number of acoustic events that were input to the cough classifier gives a coarse estimate of the number of false positives under the assumption that the number of actual coughs is a small fraction of the total amount of acoustic events, Equation (2). Also, here, the median is taken and denoted as $F_{m}$. A range $R_{\pm }$ is created by mapping to the B-scale:

(3) $$R_{\pm }=\alpha \log_{10}\left(1+\beta \left(C_{m}\pm F_{m}\right)\right).$$

The cough graph is considered true if the range $R_{\pm }$ associated with the expected FP contribution remains within the generic day-to-day variation, Section 2.2. Otherwise, if FP dominates normal variation, then the absence of clear indications of changes in cough counts may be attributable to a poor classifier instead of a patient not evidencing increased cough during exacerbation.

## 3. Results

### 3.1. Reliability

**Figure 3:**
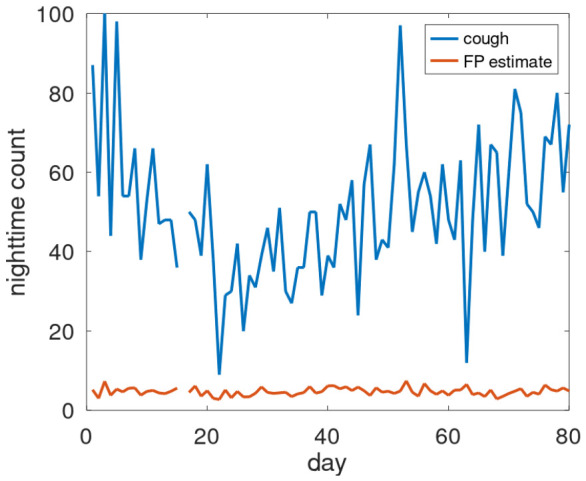
Comparison of night-time cough and false positive estimate over the monitoring period. For this patient, cough counts and FP estimates are clearly separated.

Figure 3 illustrates the trend graph of a patient with a cough classifier specificity of $s_{p}=0.994$, and the estimated FP stays below the cough trace. Since the FP estimate is low and steady, its presence does not impact the generated alerts. If an alert would be generated based on these data, we would be confident that this is not driven by artifacts in the cough data itself. It is the desired situation, but the following examples show that this is not always met.

**Figure 4:**
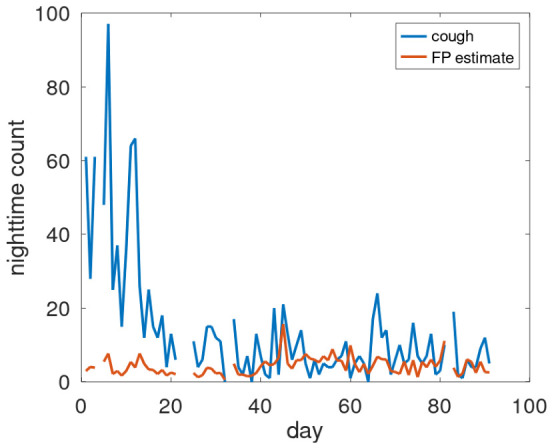
Comparison of night-time cough and false positive estimate over the monitoring period. For this patient, the lower cough counts and FP estimates are at the same level.

Figure 4 illustrates a patient where the cough is initially high but drops to a level ranging between 0 and 20 coughs/night. Overall, 12 h of monitoring with an average FP rate of 1 per hour gives an FP of around 10 and this appears to be happening for this patient. This example shows that low level cough counts are expected to be influenced by a non-perfect specificity.

**Figure 5:**
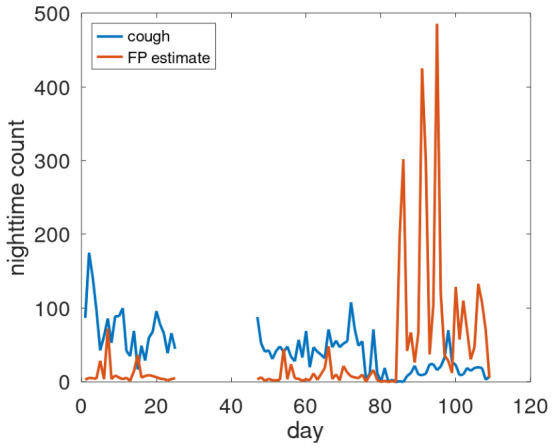
Comparison of night-time cough and false positive estimate over the monitoring period. For this patient, a sudden change in cough counts and FPs occurs around day 80.

Figure 5 presents a case where at the end of the trial (from day 80 onward) the cough count suddenly drops while the FP increases and becomes dominant. Presumably, this illustrates a changed patient behaviour: there is much more activity in the room leading to an increased number of environmental sounds and therefore an increase in the coarse FP estimate. In view of the reduced number of coughs, it is doubtful if the patient is using the bedroom for sleeping. In this last period, alerts based on the cough count become questionable.

### 3.2. Validation of Patient Screening

**Figure 6:**
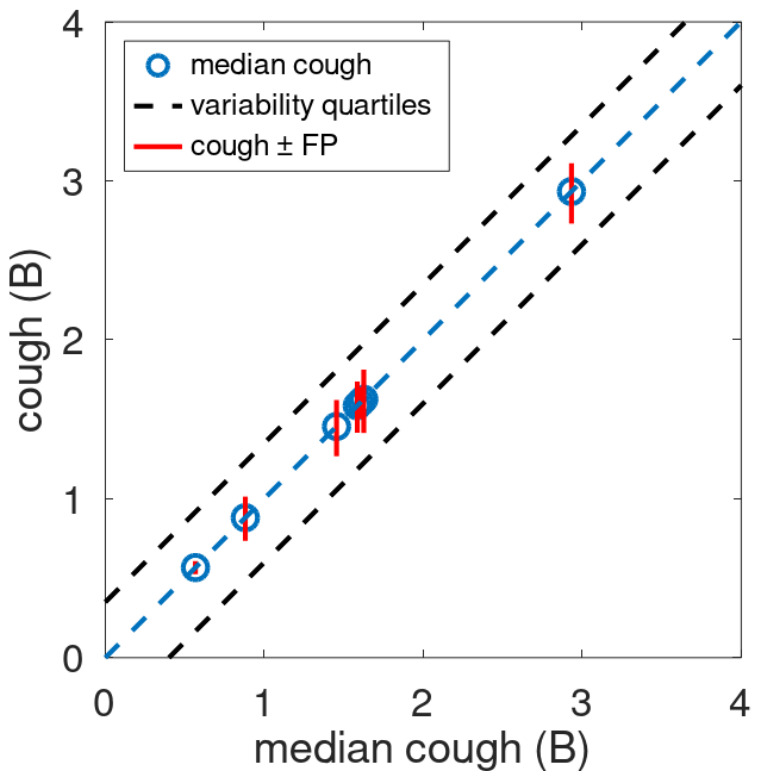
Comparison of cough counts, false alarms estimates and day-to-day variation for the seven considered COPD patients. Circles: median of detected night-time cough counts for each participant. There are four data points with *x*-coordinate between 1.45 and 1.65 B. Dashed lines: quartile ranges for day-to-day variation of night-time coughs in stable patients [28]. Red lines: range created by adding and subtracting the median estimated false positives to the detected coughs.

For the seven patients labelled as free from increased cough during exacerbation in [28], the median number of estimated FPs over the monitored nights ranged over the patients from 0.04 to 0.95 per hour. In Figure 6, the relation between detected coughs, day-to-day variation, and estimated FP is illustrated. The overall day-to-day variation of night-time cough counts in stable patients [28] is shown by its quartile range where the quartiles form straight lines parallel to the diagonal reflecting the effect of the B-scale. As crude indicator for the effect of a low specificity of the cough classifier, the estimates of the median number of false positives were added and subtracted from the median cough count and these numbers were mapped to the B-scale, Equation (3). We observe that the effect is limited: by adding or subtracting the expected false positives, the data remain well within the quartile range of the normal day-to-day variation for each patient.

## 4. Discussion

We detailed how a cough-based alert system called XACT is based on well-defined design choices for long-term cough monitoring. The choices relate to critical issues for long-term monitoring [6] and emphasize medical inference, adherence, respecting privacy and device unobtrusiveness. An overview of the main considerations is in Table 1, resulting in clear differences with other approaches, e.g., [13,33,34,35,36]. Apart from the system itself, its integration into a medical environment requires attention and testing. This involves e.g., its use in a eHealth system or virtual ward (hospitals at home) but also stratification of patients. For obvious reasons, issuing cough monitors should be restricted to patients for whom cough is a relevant biomarker of exacerbation. In case the patient is unaware of whether or not exacerbations result in increased cough, the analysis in [28] suggests this information can be extracted from demographic data and COPD Assessment Test (CAT) score. If and how this generalizes to other respiratory diseases is an open question.

The basis for a practical method for an empirical reliability check based on cough count data was outlined. The method is blind to the actual audio data and thus compatible with privacy-preserving cough monitoring systems. Rejecting or accepting cough count data based on a fixed number (e.g., one false positive per hour) of expected false positives is not a proper metric for performance: the number of acceptable false positives depends on the accuracy imposed on the signal-to-noise ratio by the pertinent application.

As a first example, cough count and expected FP traces were compared having in mind that the number of detected coughs within the considered time span should not (potentially) consist of many false positives. It was highlighted how this provides insights into unreliable cough data. The method is practical but coarse. Both of the used quantities, the number of acoustic events $N_{ae}$ and the specificity $s_{p}$, are only approximations for the actual data. The number of acoustic events is an approximation for the number of coughs, and the specificity found in the training/testing phase may not reflect the actual situation. This is because typical classifier training/testing practice involves balancing the classes (here: cough and non-cough), creating a gap with its deployment where the prevalence of coughs is (extremely) low compared to the other acoustic events (non-coughs) [26]. This also holds for the present study where the observed acoustic instances used for training were biased to the clearly audible sounds while the duller sounds were underrepresented relative to the deployment. Nevertheless, we see from Figure 5 that the number of acoustic events (which is proportional to the FP estimate) provides valuable information on the acoustic environment. It suggests that tools like acoustic event detection and topic modelling [37,38,39,40] may create soundscape interpretations to facilitate the design of reliability metrics.

In our second example, it was considered whether the manual patient screening procedure applied in an earlier paper could be explained by masking due to a high number of false positives by the cough classifier. Not only should the number of expected false positives be low relative to the actual cough count, but it should also not be the major component in the day-to-day variation. The outlined procedure shows how the data screening that was performed based on visual inspection of the data (see Figure 2) can be put on a numerical basis.

The methodology employed to compare the natural (here: daily) variation to the expected false positives is a generic method related to a signal-to-noise analysis. It may be used to signal potentially unreliable cough count estimates and may further be deployed to construct a confidence metric for inferred alerts or other medically relevant outputs. Refining and tracking the number of expected false positives is considered a crucial asset for high-quality automated cough count systems since different patients are expected to present highly different acoustic environments [26]. We note that it implies that the used specificity number is tailored to the specific acoustic environment of the patient and not a specificity from a generic or lab environment.

The study has several limitations. Firstly, the patient cohort is small and includes only COPD patients. Secondly, the collected data prohibit full analysis (e.g., annotation) due to the priority given to privacy. It makes a rigorous statistical analysis on the present data impossible. Nevertheless, the presented examples illustrate that the proposed concepts merit further investigation and validation. The proposed reliability metric assumes that the number of acoustic events is considerably larger than the number of coughs. This is backed up by the fact that the median of the number of coughs in the annotation data was 11% and this number is lower during deployment. This needs validation and more refined rules could be developed accordingly. A potential real-time reliability metric is discussed in Appendix A and could serve as a candidate in a clinical test. For clinical practice, it would mean that if the reliability metric indicates issues on a regular basis, the use of the cough monitor for this patient has to be re-considered. One could check whether the acoustic environment and/or location of the monitor can be improved or if the monitor has to be withdrawn as being non-effective.

For the moment, the number of patients and cough data is considered too limited to construct and test a system including a confidence indicator. Instead, we inspected cough and FP graphs of all patients to have a gut feeling for its effect on the alert performance reported in [29]. This exercise suggested that the data from one patient should be ignored, as the generated cough counts are high risk of being driven by false positives. This has no effect on the number of missed exacerbations or correctly generated alerts since this patient did not witness an exacerbation and no alerts were raised. For another participant, one false alert was raised which is likely caused by an increase in environmental sounds and could be spotted by a cough count reliability metric. Lastly, there is one early alert which would most likely result in a lower lead time, namely 4 days. Altogether, it means that no dramatic effects in alert performance are expected but that, with a well-designed reliability metric, adverse acoustic environments can be identified in a timely fashion, and that some false alerts may be prevented.

## 5. Conclusions

We reviewed design choices that converted a cough counter into the patient-friendly continual monitor XACT which was validated for AECOPD detection. Patient friendliness is considered quintessential for acceptance and adherence, and is achieved by unobtrusiveness, minimal burden, and privacy preservation. Acceptance by patient and caregiver is also created by high-quality performance and insights provided by the cough trends.

We reported and illustrated how the mix of information from cough counts, day-to-day variation and classifier performance can be used as a reliability metric and as a reality check: are we actually capturing cough counts? Comparing real-world traces of cough counts and FP estimates, it was illustrated how the described empirical methodology can become part of a reliability indicator within a cough counting system. In a second illustration, it was shown how counts, variability and FP estimates were used to underpin a former selection of patients having evidenced no increased cough during exacerbation. These illustrations act as a proof of concept, and the methods need further testing and validation. The outlined concepts pave the way for confidence indicators accompanying medical inferences drawn from the cough data like in the XACT system.

## Figures and Tables

**Table 1:** Design choices for the cough-based exacerbation alert system XACT. Aspects, preferred choice, reason and non-preferred options are given. The rows Classifier refers to a cough classifier with audio features as input like shown in Figure 1. The row Alert refers to the alert mechanism which is fixed and patient-independent.

Aspect	Preference	Reason	Rejected Option
Patients	stratification	performance and costs	no selection
Hardware	stationary	hassle-free, adherence	mobile or wearable
Modality	sound	off-body	acceleration
Data transfer	features	privacy	audio
Timing	night-time	low CV	daytime or 24 h
Classifier	high specificity	low cough prevalence	high sensitivity
Classifier	patient-specific	tuned to acoustic environment	generic
Alert	rule-based	insights, explainable	AI

**Figure A1:**
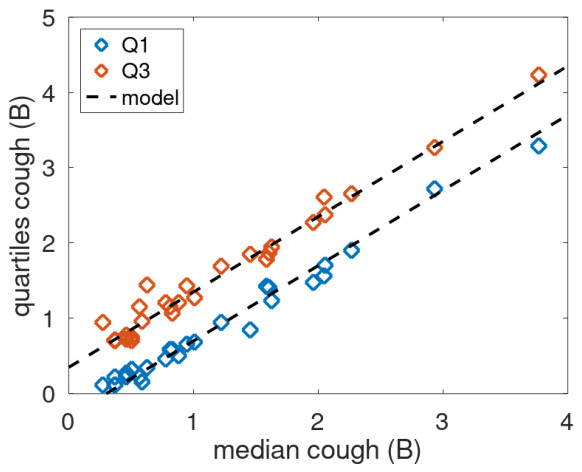
Quartile models characterising the spread of the night-time cough count distribution of stable COPD patients. Blue diamonds: first quartiles, red diamonds: third quartiles, black dashed lines: quartile model proposed in [28].

**Figure A2:**
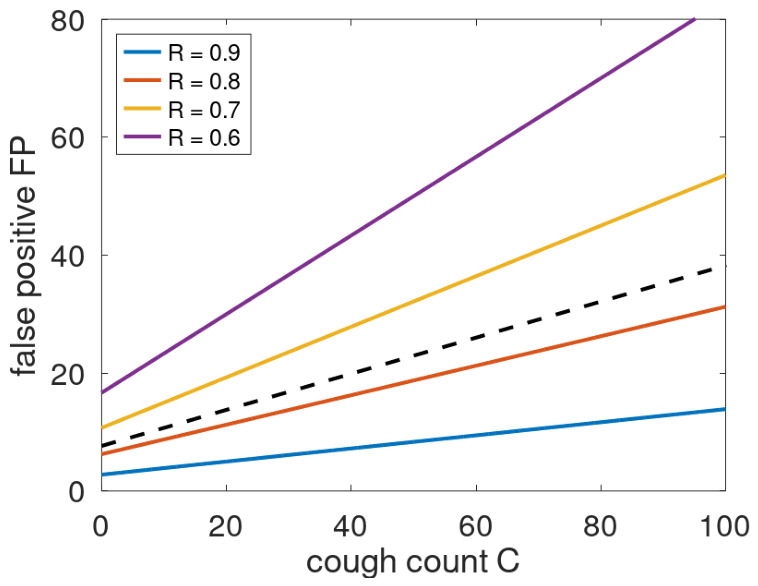
Illustration of the proposed reliability metric in the cough count (*C*)–false positive (FP) plane. The coloured lines indicate equal reliabilities according to (A2). On the dashed black lines, FP equals the generic distance between median and third quartile.

## Data Availability

The data reside at Hull York Medical School. Data are not publicly accessible in view of privacy regulations. A.H. Morice is the contact person for data requests.

## Abbreviations

The following abbreviations are used in this manuscript:

AECOPD	Acute Exacerbation of COPD
AI	Artificial Intelligence
CAT	COPD Assessment Test
COPD	Chronic Obstructive Pulmonary Disease
CV	Coefficient of Variation
FP	False Positives
TP	True Positives
XAI	eXpainable AI

## Appendix A. Reliability Metric

Consider the experimental relation between median cough count and quatile as presented in [28]. In this mapped plane, the quartiles Q1 and Q3 are modelled as lines parallel to the median; see Figure A1. This suggests that, independent of the median, the shapes of the main lobe of all distribution are very similar, except for the lowest median cough counts. This motivates us to base a reliability metric on these data.

We construct a reliability metric on a scale from 0 to 1 corresponding to lowest and highest reliability, respectively. A measurement corresponding to the median is given the highest reliability, increasing distances to the median correspond to a reduced reliability. We consider what this means for the unmapped cough counts, and how this translates to a reliability metric.

Define $M_{B}$ and $\Delta_{B}$ as the median and distance to the median on the B-scale, respectively. Similarly, we define $M_{C}$ and $\Delta_{C}$ as the median and distance to the median of the unmapped night-time cough counts *C*. It is easy to show that the straight lines with distances $\Delta_{B}$ to the median translate into a linear relation

(A1) $$\Delta_{C}=s\left(M_{C}+\gamma \right)$$

with slope $s=10^{\Delta_{B}/\alpha }-1$ and offset γ=1/β.

The most straightforward way to construct a reliability metric *R* with range [0,1] is to take

$$R=\frac{M_{C}+\gamma }{M_{C}+\gamma +\Delta_{C}}.$$

Many other expressions could serve equally well.

During deployment of the monitoring system, we have the daily numbers of night-time cough counts *C* and estimate of the false positives *F*. The measurement *C* is taken to represent $M_{C}$ while *F* quantifies the possible bias to the mean, and therefore

(A2) $$R=\frac{C+\gamma }{C+\gamma +F}$$

is introduced as the daily reliability index. Like was said, this metric is based not only on *C* and *F*, but is also firmly rooted on the day-to-day variation (i.e., the quartile model). This measure is a stochastic quantity and for practical usage it may be good to consider more robust metrics. This could be achieved by defining reliability index for data over multiple nights using robust statistics, either by considering a median over *R* or by introducing median values for *C* and *F* in the expression for *R*.

In Figure A2, Relation (A2) between the proposed reliability *R*, cough count *C* and false positives is plotted with γ=1/β=25. The linear Relation (A1) translates into straight lines for equal *R*.
